# Science and policy on endocrine disrupters must not be mixed: a reply to a “common sense” intervention by toxicology journal editors

**DOI:** 10.1186/1476-069X-12-69

**Published:** 2013-08-27

**Authors:** Åke Bergman, Anna-Maria Andersson, Georg Becher, Martin van den Berg, Bruce Blumberg, Poul Bjerregaard, Carl-Gustaf Bornehag, Riana Bornman, Ingvar Brandt, Jayne V Brian, Stephanie C Casey, Paul A Fowler, Heloise Frouin, Linda C Giudice, Taisen Iguchi, Ulla Hass, Susan Jobling, Anders Juul, Karen A Kidd, Andreas Kortenkamp, Monica Lind, Olwenn V Martin, Derek Muir, Roseline Ochieng, Nicolas Olea, Leif Norrgren, Erik Ropstad, Peter S Ross, Christina Rudén, Martin Scheringer, Niels Erik Skakkebaek, Olle Söder, Carlos Sonnenschein, Ana Soto, Shanna Swan, Jorma Toppari, Charles R Tyler, Laura N Vandenberg, Anne Marie Vinggaard, Karin Wiberg, R Thomas Zoeller

**Affiliations:** 1 Department of Materials and Environmental Chemistry, Stockholm University, SE-10691 Stockholm, Sweden; 2 Rigshospitalet, University of Copenhagen, Copenhagen, Denmark; 3 Norwegian Institute of Public Health, Oslo, Norway; 4 Utrecht University, Utrecht, The Netherlands; 5 University of California, Irvine, USA; 6 University of Southern Denmark, Odense, Denmark; 7 Karlstad University, Karlstad, Sweden; 8 Pretoria Academic Hospital, Pretoria, South Africa; 9 Uppsala University, Uppsala, Sweden; 10 Brunel University, London, UK; 11 University of Aberdeen, Aberdeen, UK; 12 Institute of Ocean Sciences, Fisheries and Oceans, Sidney, BC, Canada; 13 University of California, San Francisco, USA; 14 National Institute for Basic Biology, Okazaki, Japan; 15 Danish Technical University, Copenhagen, Denmark; 16 University of New Brunswick, Fredericton, Canada; 17 Environment Canada, Burlington, Canada; 18 Aga Khan University Hospital, Nairobi, Kenya; 19 Granada University, Granada, Spain; 20 Swedish University of Agricultural Sciences, Uppsala, Sweden; 21 Norwegian School of Veterinary Science, Oslo, Norway; 22 Stockholm University, Stockholm, Sweden; 23 ETH Zurich, Zurich, Switzerland; 24 Karolinska Institute, Stockholm, Sweden; 25 Tufts University, Boston, USA; 26 School of Medicine at Mount Sinai, New York, USA; 27 University of Turku, Turku, Finland; 28 Exeter University, Exeter, UK; 29 Tufts University, Medford, USA; 30 University of Massachusetts, Amherst, USA

**Keywords:** Endocrine disrupting chemicals, Environment, Health, Precautionary principle, Regulatory toxicology

## Abstract

The “common sense” intervention by toxicology journal editors regarding proposed European Union endocrine disrupter regulations ignores scientific evidence and well-established principles of chemical risk assessment. In this commentary, endocrine disrupter experts express their concerns about a recently published, and is in our considered opinion inaccurate and factually incorrect, editorial that has appeared in several journals in toxicology. Some of the shortcomings of the editorial are discussed in detail. We call for a better founded scientific debate which may help to overcome a polarisation of views detrimental to reaching a consensus about scientific foundations for endocrine disrupter regulation in the EU.

## Commentary

“Common sense is the collection of prejudices acquired by age eighteen”

- Albert Einstein

As experts and practitioners of endocrine disrupter research, several of whom were invited to prepare some recent international status reports of the topic [1-4], we, the authors, would like to comment on the recent editorial “Scientifically unfounded precaution drives European Commission’s recommendations on EDC regulation, while defying common sense, well-established science and risk assessment principles” by Dietrich et al. [5].

We are concerned that the Dietrich editorial appears to be intended as an intervention designed to impact imminent decisions by the European Commission concerning endocrine disrupting chemicals (EDCs), countering the views recently expressed by the 129 signatories of the Berlaymont Declaration on endocrine disrupters [6] and by the Collegium Ramazzini [7]. Given the prominent nature of the authors as members of several EU scientific committees and the importance of these decisions, we would have expected a more accurate analysis of the situation. In contrast, the editorial confuses and conflates several aspects of the current debate that are important to clarify. In general, their fears appear to be founded on a ‘common sense’ that largely ignores the continued efforts of many scientific expert groups at European and international level as well as the expertise and competence of European decision makers.

First, in describing endocrine systems as “… play[ing] a fundamental role in the physiological response to changes in the environment with the aim of keeping an organism’s response within the homeostatic space” Dietrich et al. seek to define the endocrine system in overly simplistic terms to reduce the task of identifying endocrine disruption to making distinctions “between those effects that are within this adaptive range and effects that go beyond the boundaries of this space and thus can be called adverse” [5]. It is perplexing that editors of international toxicology journals seem to be unaware of the fact that endocrine systems also have a programming role during development, and that disruption of these programming events leads to irreversible effects that go far beyond disturbances of homeostasis [1]. Such phenomena (for example disruption of androgen action in fetal life and the malformations that arise from this) have been described for decades in the scientific literature and provide some of the cause for concerns about endocrine disrupting chemicals. These and other clearly demonstrated cases necessitate the identification of specific windows of vulnerability and this poses considerable challenges to established toxicity testing paradigms, all of which Dietrich et al. [5] ignore.

## Thresholds and no thresholds

Dietrich et al. [5] claim that the “currently drafted EU framework” is based on an *a priori* default assumption of no thresholds for regulating endocrine disrupters, but no document is referenced to substantiate this claim. The latest publicly available document from the European Commission is the Report of the Endocrine Disrupters Expert Advisory Group (ED EAG) published by Directorate General Joint Research Centre (JRC) [8] which is intended to provide the underpinnings of the future EU regulatory framework for endocrine disrupters. The Report was prepared by an expert group comprised of 43 members from competent authorities representing 19 member countries of the European Union as well as other stakeholders including environment and health, NGOs and the industry-funded scientific association, ECETOC. The circumstances that led up to this Report are at odds with the claim by Dietrich et al. [5] that the proposed regulatory framework “is based on virtually complete ignorance of all well-established and taught principles of toxicology and pharmacology, of opinions raised by the European Commission’s own competent expert authority (…), and of critical statements made by EU member states…”. In the JRC document [8], no reference is made to a presumed *a priori* assumption of no thresholds for endocrine disrupters.

From a scientific standpoint, the issue of the existence of a threshold for endocrine disrupters and other non-genotoxic toxicants remains under debate. As Dietrich et al. [5] rightly point out, absence of effect cannot be statistically demonstrated in an experimental setting. It derives from this that regardless of the mode-of-action and the existence or non-existence of a mechanistic threshold, such a threshold cannot be demonstrated experimentally. If science prides itself in the robustness of its experimental approach to evidence, it should be stressed that the current argument can be modelled or theorised upon, but cannot currently be definitively experimentally tested. Regarding the claim that “…the weight of evidence (…) clearly demonstrates the presence of threshold for non-genotoxic compounds including EDCs…”, Dietrich et al. [5] ignore that this evidence is far from established. In international toxicology journals, not under the editorship of Dietrich et al. [5], widely accepted biometrical and mathematical principles about the impossibility of establishing thresholds at the level of populations, independent of the status of the chemicals in terms of genotoxicity or non-genotoxicity have been elaborated [9,10].

## Adversity of effects

It is also unclear where the claim by Dietrich et al. [5] that “the currently drafted EU framework for EDCs foresees a priori regulation of agents that may show presumably endocrine-mediated effects in some experimental system (*in vitro*, *in silico*, *in vivo*…)” derives from. The JRC report clearly states that for a substance to be identified as an endocrine disrupter, evidence not only of an endocrine mode-of-action but also of an adverse effect is required, as well as some plausible link between mode-of-action and adversity. This is consistent with the widely accepted IPCS definition [11] of endocrine disrupters which the JRC report accepted.

Concerning assays or endpoints that would be considered adequate for assessments of evidence of adverse effects, the JRC report makes detailed reference to level 4 or level 5 of the assays included in the OECD Conceptual Framework for the assessment of endocrine disrupters. This framework is the result of expert efforts over many years [12]. Although many endpoints relevant to endocrine disruption are not included in the OECD study guidelines, the tests that form part of the current framework are validated, robust, reproducible methods that have been tested in many laboratories before approval to ensure consistent, valid results that are also recognised worldwide under the OECD Mutual Acceptance of Data. These can hardly be qualified as “irrelevant tests” as Dietrich et al. [5] have done.

## *A priori* assumption of human relevance

Referring to a statement by the European Commission (again not referenced) that “relevance of the data to humans should be assumed in the absence of appropriate data demonstrating non-relevance”, Dietrich et al. [5] declare: “The mere statement demonstrates the lack of attention paid by the European Commission to the weight of scientific evidence that clearly demonstrates the presence of a threshold for non-genotoxic compounds including EDC”. Here, the authors conflate the statistical impossibility of demonstrating the absence of effects (and thresholds) with the issue of demonstrating human relevance of toxicity data derived from testing on animals. In doing so they reveal ignorance of important risk assessment principles elaborated in an IPCS Framework document [11] for assessing the human relevance of non-cancer endpoints [13]. The default assumption under that framework is of human relevance, unless there is evidence of toxicodynamic or toxicokinetic differences between the animal test species and humans that shows that the effect seen in animals is not expected to occur in humans. The applicability of that default assumption was tested through a number of case studies [13]. The alternative *a priori* assumption (that effects seen in animals are not relevant for humans) would be unworkable and would undermine the sense of conducting toxicological testing in animals at all.

## “Scientifically unfounded precaution”, and the distinction between hazard assessment and risk management

The most worrying aspect of the editorial by Dietrich et al. [5] is the blurring of the border between what constitutes science and what belongs to the realm of political, societal and democratic choices.

The Precautionary Principle is enshrined in European Law in the EC Treaty as well as in International Law [14]. This principle was elaborated at the 1992 Rio Conference on the Environment and Development, during which the Rio Declaration was adopted. Principle 15 states that: “in order to protect the environment, the precautionary approach shall be widely applied by States according to their capability. Where there are threats of serious or irreversible damage, lack of full scientific certainty shall not be used as a reason for postponing cost-effective measures to prevent environmental degradation” [14]. Defined in this way, the precautionary principle is a legal concept for addressing scientific uncertainty, and not a scientific concept. Its interpretation and application is a matter for politicians and lawyers. The state of the science on endocrine disruption has been reviewed and summarised in several recent reports published by the UNEP/WHO or commissioned by the European Commission [1,2,8,15]. Already over 10 years ago, it was concluded that the state of the science justified regulatory action [13]. Decisions as to what kind of action may be justified by the level of available evidence and proportionate to the potential risks is a matter for politicians and risk managers, and not the exclusive domain of scientists. Yet Dietrich et al. [5] express strong reservations regarding the application of EU law but do not engage with the scientific basis for concern, or with widely published scientific evidence.

In contrast, the JRC report [8] made a clear distinction between hazard identification and characterisation on the one hand, which they considered within the remit of their expertise, and risk management on the other.

Scientific truths about endocrine disruption as a phenomenon resulting from disturbances of the programming effects of the endocrine system during development seem to have been ignored by Dietrich et al. [5]. It is to be hoped that this editorship of international toxicological journals will be able to engage in a better founded scientific debate which may help to overcome a polarisation of views detrimental to reaching a consensus about scientific foundations for endocrine disrupter regulation in the EU.

## Abbreviations

EC: European communities; ECETOC: European centre for ecotoxicology and toxicology of chemicals; ED EAG: Endocrine disrupter expert advisory group; EDC: Endocrine disrupting chemical; EU: European union; IPCS: International programme for chemical safety; NGO: Non-governmental organisation; OECD: Organisation for economic cooperation and development.

## Competing interests

All authors declare that they have no competing interests. Several of the authors were invited by the European Commission and UNEP/WHO, as scientific experts, to prepare some recently published international reports on state of the science of endocrine disrupters.

## Authors’ contributions

A core croup of the authors first drafted the manuscript and circulated it for comments. All authors contributed actively to the revision of the draft. All authors approved the final version.
